# Comparison of the dose escalation potential for two hypofractionated radiotherapy regimens for locally advanced pancreatic cancer

**DOI:** 10.1016/j.ctro.2019.03.001

**Published:** 2019-03-08

**Authors:** Jenny Bertholet, Arabella Hunt, Alex Dunlop, Thomas Bird, Robert A. Mitchell, Uwe Oelfke, Simeon Nill, Katharine Aitken

**Affiliations:** a Joint Department of Physics, The Institute of Cancer Research and The Royal Marsden NHS Foundation Trust, 15 Cotswold Road, London SM2 5NG, UK; b The Institute of Cancer Research, London SM2 5PT, UK; c The Royal Marsden NHS Foundation Trust, Downs Rd, Sutton SM2 5PT, UK; d The Bristol Cancer Institute, Bristol BS2 8ED, UK

**Keywords:** Pancreatic cancer, SBRT, Hypofractionation, Dose escalation, Treatment planning

## Abstract

**Objectives:**

To determine the potential for dose escalation to a biological equivalent dose BED_10_≅100 Gy in hypofractionated radiotherapy for locally advanced pancreatic cancer (LAPC).

**Materials and methods:**

Ten unselected LAPC patients were retrospectively included in the study. Two fractionation regimens were compared (5 and 15 fractions). The aim was to cover 95% of the Planning Target Volume (PTV) with a BED_10_ = 54 Gy (base dose = 33 Gy in 5 fractions, 42.5 Gy in 15 fractions) whilst respecting organs-at-risk (OAR) constraints. Once the highest PTV coverage was achieved dose escalation to a BED_10_≅100 Gy (escalated dose = 50 Gy in 5 fractions, 67.5 Gy in 15 fractions) was attempted, limiting the PTV maximum dose to 130% of the escalated dose.

**Results:**

In 5 fractions, 95% PTV coverage by both base and escalated doses could be achieved for one patient with PTV more than 1 cm away from OAR. 95% and 90% PTV coverage by the base dose was achieved in one and two patients respectively. In all other patients, coverage even by the base dose had to be compromised to comply with OAR constraints. In 15 fractions, 95% PTV coverage by the base dose was feasible for all patients except one. Dose escalation allowed improvement in target coverage by the base dose in both fractionation regimen whilst covering a sub-volume of the PTV with a BED_10_≅100 Gy. Both fractionation schemes were equivalent in terms of dose escalation potential.

**Conclusion:**

LAPC patients with OAR close to the PTV are generally not eligible for hypofractionation with dose escalation. However, this planning study shows that it is possible to cover PTV sub-volumes with a BED_10_≅100 Gy in addition to delivering a BED_10_ = 54 Gy to 90–95% of the PTV as commonly prescribed to this population. Combined with an adaptive approach, this may maximize PTV coverage by a high BED on days with favourable anatomy.

## Highlights

• The dose for hypofractionated RT for LAPC is limited by the proximity of OAR.
• Uniform target coverage by a BED of 54 Gy is easier in 15 than in 5 fractions.
• But the dose escalation potential to a BED of 100 Gy is similar in 5 or 15 fractions.
• Larger tumour sub-volumes may receive a high dose on days with favourable anatomy.

## 1 Introduction

In 2017, pancreatic cancer was predicted to be the 3rd most common cause of cancer death in the United States (US) with a 5 year survival rate of only 8% [1]. At diagnosis, 30% of patients present with locally advanced, unresectable disease [2].

Worldwide, the optimal treatment for locally advanced pancreatic cancer (LAPC) is controversial with conflicting results from phase 3 clinical trials on the survival benefit of standard fractionation chemoradiation (CRT) compared to chemotherapy alone [3], [4], [5], [6].

In recent years, hypofractionation and stereotactic body radiotherapy (SBRT) have been increasingly investigated for the treatment of LAPC. Characterized by high dose per fraction and highly conformal dosimetry, these offer the convenience of shorter overall treatment time, reduced time off full dose systemic therapy and potentially, improved local control. Yet, the close proximity of radiosensitive organs at risk (OAR) has led to so-called ‘low dose’ SBRT becoming a favoured approach [7]. Low rates of toxicity with a 1 year local control of 78% have been reported for dose regimens such as 33 Gy in 5 fractions [8]. This corresponds to a biological equivalent dose (BED_10_) of 54.8 Gy, slightly lower than conventional regimens delivering a BED_10_ of 60–64 Gy [4], [9].

Although there have been no randomized control trials comparing CRT to SBRT, a systematic review of 19 trials suggests a median survival of 17 months with acceptable toxicity when using an SBRT approach [10]. This advantage is supported by a cancer database review from the US suggesting an improvement in overall survival in patients undergoing SBRT compared to other types of radiotherapy [11]. It should be noted that within these studies, heterogeneity of treatment regimens was significant.

In an attempt to optimise the use of radiotherapy in LAPC, recent interest has focussed on the role of dose escalated treatments. A retrospective review by Krishnan et al.[12] suggests that dose escalation above a BED_10_ of 70 Gy increases survival from 15 months to 17.8 months (p = 0.03), whilst biophysical models suggest that this benefit can be further extended through dose escalation up to a BED_10_ of 100 Gy [13]. Other studies have shown similar positive results when escalating above traditional thresholds [14], [15]. In the majority of cases, dose escalation has been achieved using SBRT regimens (≤5 fractions) for targets smaller than 5 cm in diameter [15] or located >1 cm away from gastrointestinal (GI) OAR [12]. Dose escalation to sub-volumes of the targets (also known as simultaneous integrated boost or SIB) was also proposed for tumours without duodenal involvement [16], [17]. However, any form of dose escalation is challenging for lesions in close proximity to OAR. For patients with unfavourable anatomy, moderate hypofractionation (e.g. 15 fractions) may facilitate dose escalation whilst maintaining OAR constraints. However, dose escalated moderate hypofractionation has not yet been fully evaluated in patients with OAR located less than 1 cm away from the PTV.

The aim of this study is to investigate the potential for dose escalation using hypofractionated radiotherapy for a group of LAPC patients with a varying degree of OAR proximity and evaluate if there is a dosimetric benefit of moderate hypofractionation in 15 fractions compared to 5 fractions in achieving a BED_10_≅100 Gy.

## 2 Materials and methods

### 2.1 Patients

Following approval from the local institutional review board, CT planning scan images for ten consecutive LAPC patients treated with radical CRT (54 Gy in 30 fractions) between May 2016 and November 2017 at our institution were re-contoured. All patients had prospectively given consent for their anonymised data sets to be used for research purposes. The Planning Target Volume (PTV) was defined as the Gross Tumour Volume (GTV) plus a 5 mm isotropic margin, assuming treatment delivery in breath-hold using Active Breathing Coordinator (ABC) [18], [19]. The duodenum, stomach, small bowel, large bowel, liver, kidneys, and spinal cord were identified as OAR and delineated.

### 2.2 Fractionation and dose escalation

Two fractionation regimens (5 and 15) were compared in this study. The base prescription dose was chosen to be equivalent to a BED_10_ = 54 Gy, based on an SBRT prescription of 33 Gy in 5 fractions [8]. In 15 fractions, the base prescription was 42.5 Gy. The aim was to cover 95% of the PTV with the prescription dose for both fractionations with maximum dose to the PTV limited to 130% of the prescription dose. The OAR constraints (Table 1) were prioritized over target coverage. The OAR constraints for the two fractionations were taken from currently recruiting trial protocols [20], [21]. In cases where 95% PTV coverage to the prescription dose could not be achieved, the highest achievable dose coverage whilst respecting the OAR constraints was reported.

**Table 1:** OAR constraints for 5 and 15 fractions.

Organ	5 fractions [20]	15 fractions [21]
Duodenum	V_33Gy_ ≤ 0.5 cc	V_45Gy_ ≤ 0.5 cc
Stomach	V_33Gy_ ≤ 0.5 cc	V_40Gy_ ≤ 0.5 cc
Small bowel	V_33Gy_ ≤ 0.5 cc	V_45Gy_ ≤ 0.5 cc
Large bowel	V_33Gy_ ≤ 0.5 cc	V_48Gy_ ≤ 0.5 cc
Liver	D_mean_ ≤ 20 Gy and V_15Gy_ ≤ 700 cc	D_mean_ ≤ 22 Gy
Kidneys (combined)		D_mean_ ≤ 12 Gy
Kidneys (each)	D_mean_ ≤ 12 Gy and D_67%_ ≤ 8 Gy	V_12Gy_ ≤ 10%*
Spinal cord	V_25Gy_ < 0.5 cc	V_35Gy_ ≤ 0.5 cc

* If solitary kidney or if D_mean_ >12 Gy for one kidney.

Once this was achieved, dose escalation to a BED_10_≅100 Gy was attempted. Dose prescriptions for the escalated plans were 50 Gy in 5 fractions (BED_10_ = 100 Gy) and 67.5 Gy in 15 fractions (BED_10_ = 98 Gy). The aim was to maximize the volume receiving the escalated dose whilst maintaining or increasing PTV coverage by the base dose (BED_10_ = 54 Gy). The maximum allowable dose to the PTV was 130% of the escalated dose.

### 2.3 Treatment planning technique

Volumetric Modulated Arc Treatment (VMAT) plans were designed according to our institutional standard for treatment on an Elekta linac with an Agility multi-leaf collimator (MLC) (Elekta AB, Stockholm, Sweden) with a single 6 MV Flattening-Filter Free (FFF) arc from 179° to 181° gantry rotation in Raystation 6.99 (RaySearch Laboratories, Stockholm, Sweden). The collimator rotation was set at 5° and the maximum MLC leaf speed was constrained at 0.6 cm/deg to limit plan modulation and increase treatment efficiency. The delivery time was restricted to approximately 100 s, such that treatment would be deliverable in 5–10 breath holds depending on patient compliance.

### 2.4 Data analysis

The achievable target coverage by the prescription dose was reported for all plans as well as the coverage by the base dose for the dose escalated plans. The Paddick conformity index, ${\mathrm{CI}}_{\mathrm{PADDICK}}$ [22] was calculated for all plans.

The overlapping volume between the PTV and duodenum, stomach, small bowel or large bowel relative to the PTV volume was calculated as:

(1) $${\mathrm{PTV}}_{\mathrm{OAR}}=100\frac{\mathrm{volume}(PTV\cap OAR)}{\mathrm{volume}(PTV)}$$

where OAR=(duodenum∪stomach∪smallbowel∪largebowel).

However, in some cases with little overlap, the OAR are abutting the target, therefore also limiting PTV coverage. In order to quantify this effect, the OAR proximity volume was calculated as the volume of OAR in the 1 cm periphery of the PTV divided by the total PTV volume:

(2) $${\mathrm{OAR}}_{\mathrm{prox}}=100\frac{\mathrm{volume}({\mathrm{PTV}}_{\mathrm{rim}}\cap OAR)}{\mathrm{volume}(PTV)}$$

where ${\mathrm{PTV}}_{\mathrm{rim}}=\left(PTV+1cm\right)⧹PTV$.

The Pearson’s correlation coefficient was calculated between PTV volume, ${\mathrm{PTV}}_{\mathrm{OAR}}$ or ${\mathrm{OAR}}_{\mathrm{prox}}$and target coverage to investigate the effect of target size and OAR overlap or proximity on achievable coverage (significance level of 5%).

## 3 Results

Patients’ PTV volumes, limiting OAR, ${\mathrm{PTV}}_{\mathrm{OAR}}$ and ${\mathrm{OAR}}_{\mathrm{prox}}$ volumes are listed in Table 2.

**Table 2:** Target volume, dose limiting OAR and overlapping/abutting volumes.

Patient	PTV volume [cc]	GTV volume [cc]	Closest OAR (volume overlap with PTV [cc])	Duodenum, small bowel, large bowel and stomach:	
				${\mathrm{PTV}}_{\mathrm{OAR}}$[%] (Eq. (1))	${\mathrm{OAR}}_{\mathrm{prox}}$[%] (Eq. (2))
1	10.82	2.24	–	0.00	1.4
2	52.96	19.07	Duodenum (5.97)	11.27	48.73
3	33.41	10.41	Duodenum (2.02)	6.05	68.12
4	52.27	19.87	Duodenum (8.7)	16.64	53.97
5	92.51	43.44	Small bowel (0.56)	0.84	29.02
			Stomach (0.22)		
6	196.73	103.57	Stomach (9.44)	7.04	43.35
			Small bowel (1.73)		
			Large bowel (2.68)		
7	77.94	35.29	Duodenum (5.2)	6.67	33.64
8	96.44	42.46	Duodenum (9.45)	10.07	56.25
			Small bowel (0.17)		
9	57.52	23.69	Duodenum (5.64)	9.89	62.52
10	222.81	134.01	Duodenum (7.77)	3.85	23.90
			Large bowel (0.81)		

**Fig. 1:**
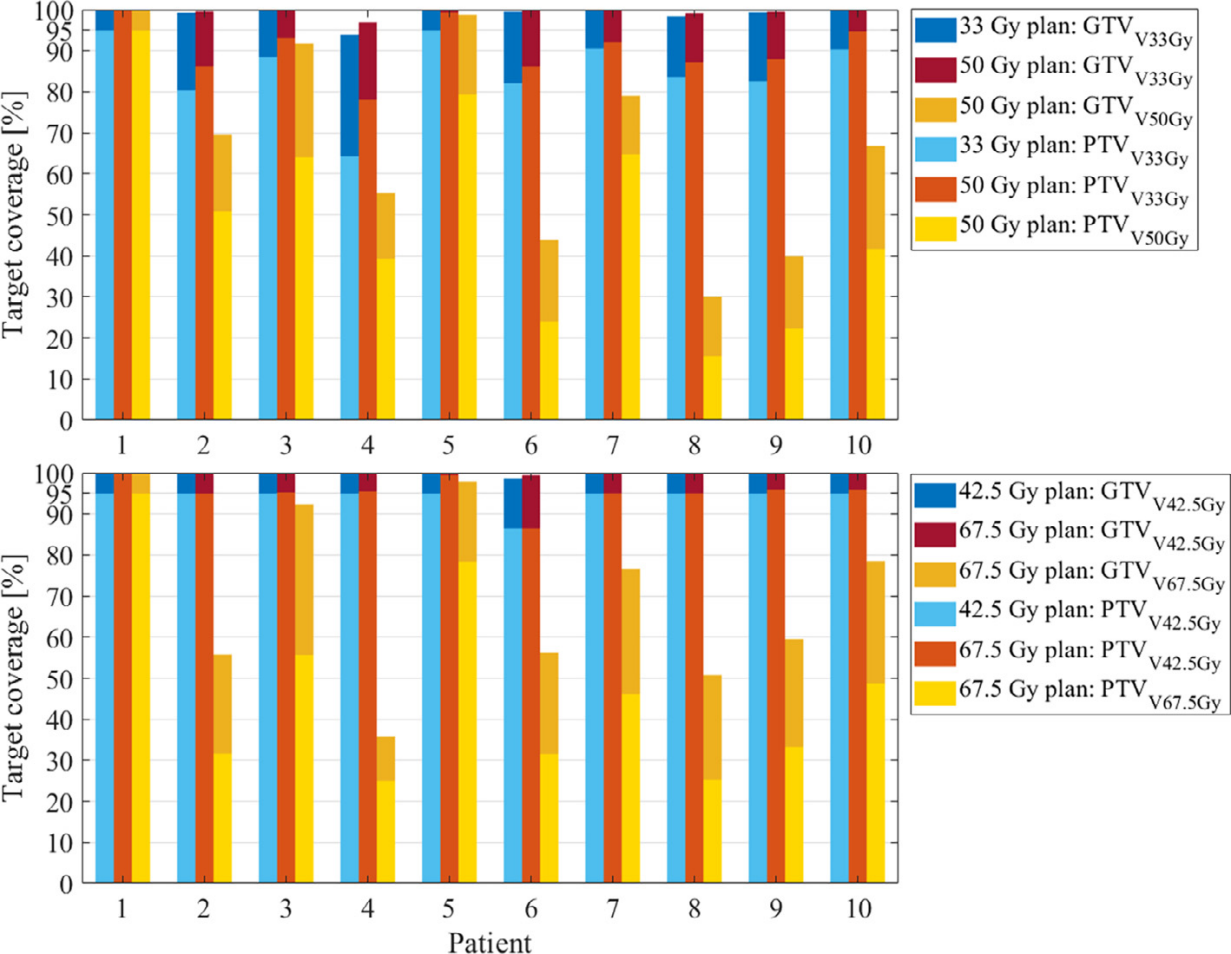
Target coverage by the base (blue and red) and escalation (yellow) dose in 5 (top) or 15 (bottom) fractions. (Blue bars indicate the base plans. Red and yellow bars indicate dose escalation plans. Darker bars indicate the GTV and lighter bars indicate the PTV). (For interpretation of the references to colour in this figure legend, the reader is referred to the web version of this article.)

**Fig. 2:**
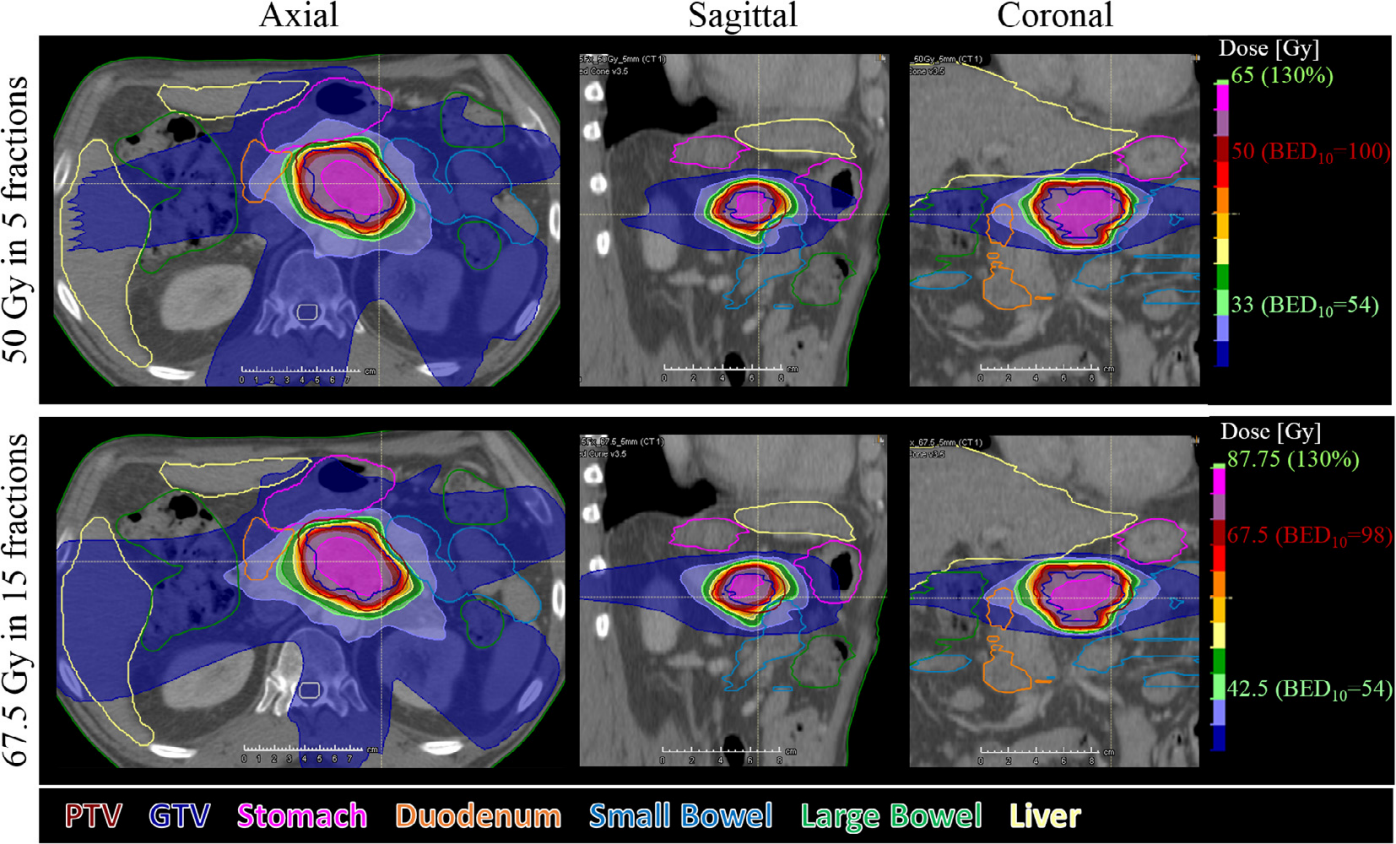
Dose distribution for the escalated plan in 5 fractions (top) and 15 fractions (bottom) for patient 5. Target coverage is equivalent in the 2 fractionation regimen.

In 5 fractions, 95% PTV coverage by the base dose was achieved for patient 1 (PTV_OAR_ = 0%) with ${\mathrm{CI}}_{\mathrm{PADDICK}}$ = 0.85 (Fig. 1) and for patient 5 (PTV_OAR_ = 0.84%) with ${\mathrm{CI}}_{\mathrm{PADDICK}}$ = 0.90 (Fig. 2, top). For the other patients, coverage was compromised in order to comply with OAR constraints (mean ± SD PTV V_33Gy_ = 83 ± 8%). PTV V_33Gy_ was greater than 90% for patients 7 and 10 (${\mathrm{CI}}_{\mathrm{PADDICK}}$ of 0.81 and 0.77 respectively). In the dose escalated plans, 95% PTV coverage by the escalated dose was also achieved for patient 1 with ${\mathrm{CI}}_{\mathrm{PADDICK}}$ = 0.85. For patient 5, PTV V_33Gy_ was increased to 99%, however, V_50Gy_ was limited to 79% (${\mathrm{CI}}_{\mathrm{PADDICK}}$ = 0.77). For the other eight patients, PTV coverage by the base dose was slightly improved (Fig. 1 top) (mean ± SD PTV V_33Gy_ = 88 ± 5%) and the mean ± SD PTV coverage by the escalated dose was PTV V_50Gy_ = 40 ± 19%.

In 15 fractions, 95% PTV coverage by the base dose was achieved for nine out of ten patients (Fig. 1 bottom) with mean ± SD${\mathrm{CI}}_{\mathrm{PADDICK}}$ = 0.89 ± 0.03. For patient 6 who had significant overlap between the PTV and the stomach, PTV V_42.5Gy_ was 86% with ${\mathrm{CI}}_{\mathrm{PADDICK}}$ = 0.83. For the dose escalated plans, 95% PTV coverage was achieved for patient 1 (${\mathrm{CI}}_{\mathrm{PADDICK}}$ = 0.84). For the other patients, dose escalation to the PTV had to be compromised in order to comply with OAR constraints. The mean ± SD PTV coverage by the escalated dose was PTV V_67.5Gy_ = 42 ± 17% and the PTV volume receiving the base dose was greater than 95% for all patients except patient 6 (PTV V_42.5Gy_ = 86%) (Fig. 1 bottom).

**Fig. 3:**
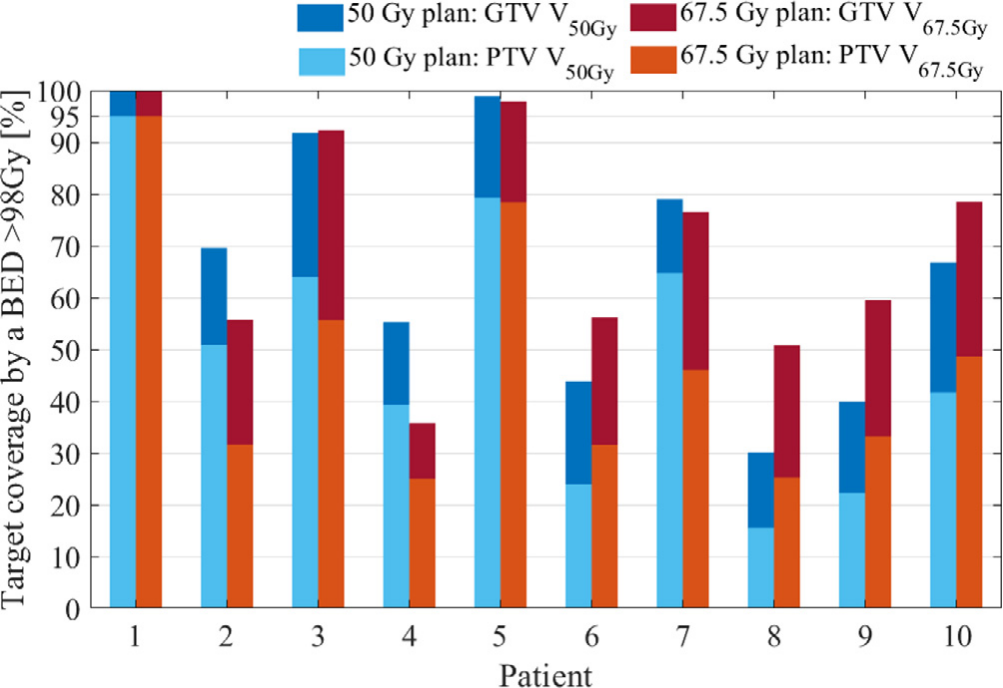
Potential for dose escalation in 5 (blue bars, equal to yellow bars in Fig. 1 top) and 15 (red bars, equal to yellow bars in Fig. 1 bottom) fractions with a BED≅100 Gy. (For interpretation of the references to colour in this figure legend, the reader is referred to the web version of this article.)

**Fig. 4:**
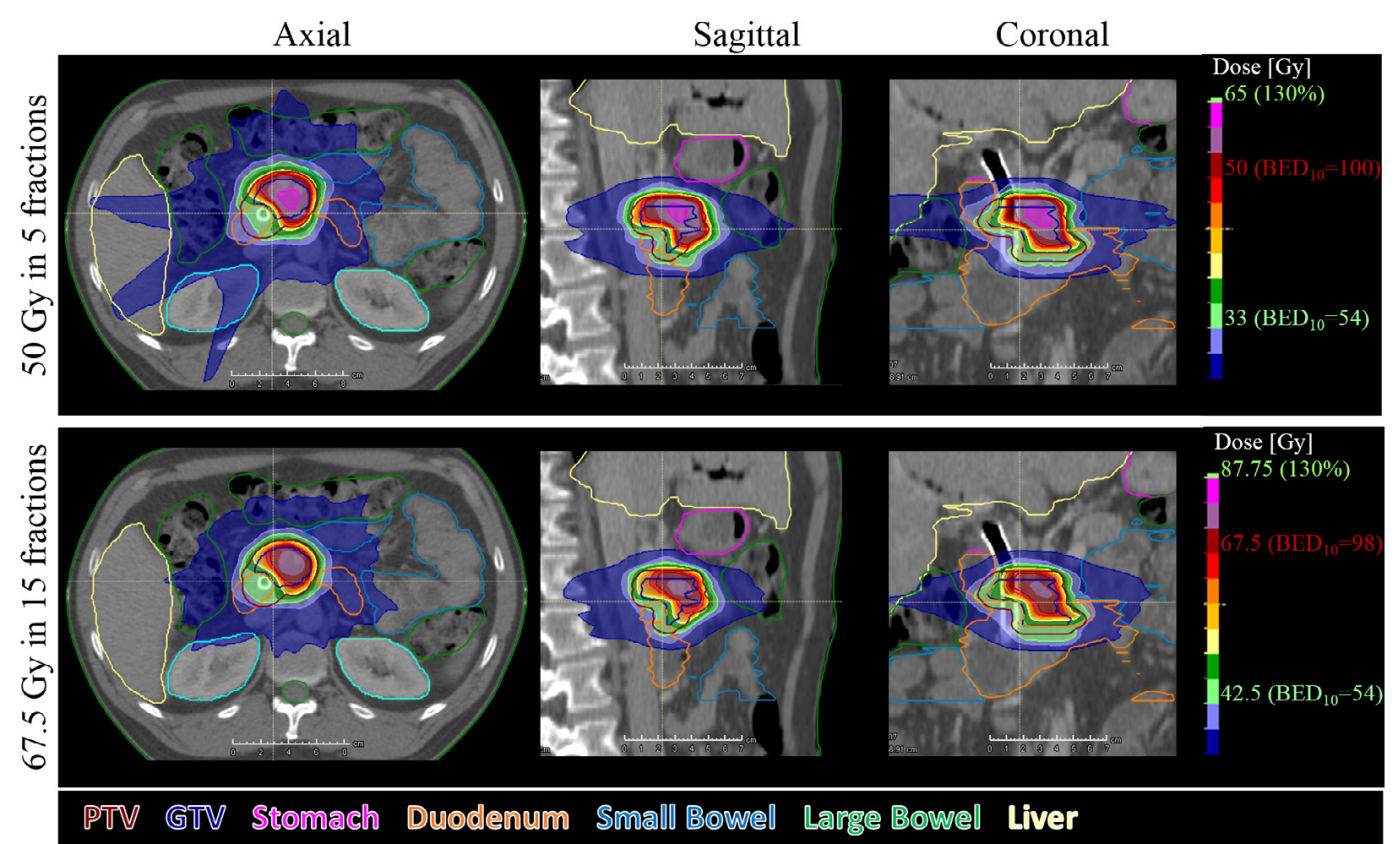
Dose distribution for the escalated dose plan in 5 fractions (top) and 15 fractions (bottom) for patient 2. PTV Coverage by the base dose (light green) is higher in 15 fractions (95%) than in 5 fractions (86%). However, PTV coverage by the escalated dose (dark red) is higher in 5 fraction (51%) than in 15 fraction (32%). (For interpretation of the references to colour in this figure legend, the reader is referred to the web version of this article.)

The PTV volume receiving the escalated dose was greater for 5 fractions than 15 fractions for four patients, greater for 15 fractions than 5 fractions for four patients and equivalent for both fractionation for patient 1 and 5 (Fig. 3). Fig. 4 shows the dose distributions for patient 2 which illustrates the better coverage by the base dose in 15 fractions (bottom) despite a better coverage by the escalated dose in 5 fractions (top). Plan delivery time was below 100 s for all plans except one plan with a delivery time of 113 s.

PTV volume, ${\mathrm{PTV}}_{\mathrm{OAR}}$ and ${\mathrm{OAR}}_{\mathrm{prox}}$ are reported in Table 2. As expected, there was a strong, negative correlation between overlap metrics and PTV coverage by the base dose in 5 fractions and by the escalated dose in both fractionation regimens (correlation below −0.64 in all cases, p < 0.05). The correlation between PTV volume and target coverage was low and non-significant for all fractionations.

## 4 Discussion

The benefit of a high BED for local control in LAPC has been previously demonstrated in tumours smaller than 5 cm and/or more than 0.5 cm away from GI OAR [7], [12], [15], [23]. However, dose escalation is challenging when the anatomy is less favourable, especially using a 5 fraction SBRT approach. Moderate hypofractionation (e.g. 15 fractions) was investigated as a possible solution to this problem. In this study we compared the potential for dose escalation to a BED_10_≅100 Gy in 5 or 15 fractions in an unselected group of LAPC patients representing a real-world population.

The patients presented a range of PTV_OAR_ and OAR_prox_ volumes leading to varying PTV coverage (Fig. 1, Fig. 2). In 5 fractions, 95% PTV coverage by the escalated dose was achievable for one patient and 95% coverage by the base dose was achieved for two patients with PTV_OAR_ <1% indicating that patients with favourable anatomy may be treated to 50 Gy in 5 fractions as described in other studies [7], [12], [15], [23]. Two other patients had 90% PTV coverage by the base dose.

In 15 fractions, 95% PTV coverage by 42.5 Gy was feasible for all patients except one with overlap between the PTV and the stomach. The stomach had a more conservative dose constraints than the other OARs with no more than 0.5 cc receiving 40 Gy, whereas dose constraints to the other OARs was higher than the prescription dose of 42.5 Gy.

Our initial hypothesis was that dose escalation would be easier in 15 than in 5 fractions leading to larger sub-volumes of the PTV receiving the escalated dose in 15 fractions. This hypothesis was not verified and treatment plans for 5 and 15 fractions showed equivalent performances over our cohort with a possible advantage of 15 fractions for larger tumours (patients 6, 8 and 10) although no significant correlation was found between target coverage and PTV volume. A 5-fraction regimen offers the advantage of a reduced overall treatment time and is less labour intensive in case of daily adaption. However, due to the risk of inter- and intrafractional anatomical changes, advanced adaptive technique should be used to ensure that OARs do not enter a high dose region. A 15-fraction regimen is more forgiving to interfractional errors, allows for concomitant chemotherapy and may be dosimetrically advantageous for large tumours. The longer overall treatment time may allow for response-based adaption. However, there is currently no prospective data for dose escalated 15 fraction regimens, and therefore it remains to be seen whether toxicity rates including rates of lymphopenia and immunosuppression differ between the two regimens.

Despite the similar potential for dose escalation, target coverage by the base dose was more easily achieved in 15 fractions than in 5 fractions (Fig. 3, Fig. 4). It is yet unknown whether a high dose to sub-volumes of the GTV is preferable to more complete coverage of the entire GTV by a lower dose. In the latter case, the required minimum sub-volume coverage and minimum boost dose should be investigated in prospective trials.

The need to compromise the dose escalation to the PTV to comply with OAR constraints in dose escalated plans indicates that a heterogeneous dose distribution is most likely the best option to achieve a BED_10_≅100 Gy for LAPC patients in hypofractionated regimens.

Previous studies on heterogeneous dose prescription in pancreas SBRT aiming at boosting sub-volumes of the PTV were limited to patients without duodenal involvement [16], [17]. Wo et al. investigated the effect of dose painting to deliver a higher dose (BED_10_ = 71.2 Gy) in 28 fractions to regions of vessels involvement with the aim to convert unresectable and borderline resectable cases to resectable [16]. They found dose painting to be feasible and well tolerated and 37% of the patients were able to proceed to resection. Shaib et al investigated the use of a simultaneous integrated boost (SIB) to the posterior margin (PM) of borderline resectable patients [17]. The PM dose ranged from 36 to 45 Gy in 3 fractions (BED_10_ from 79.2 to 112.5 Gy) whilst the dose to the PTV was either 30 or 36 Gy (BED_10_ = 60 or 79.2 Gy). Dose limiting toxicity was not reached and eight of thirteen patients had R0 resection after SBRT.

Gkika et al. used a de-escalation approach using simultaneous integrated protection (SIP). This involved reduced dose to PTV sub-volumes overlapping with OAR protection volumes [24]. They observed a favourable toxicity profile without compromise in tumour control.

In the present study, heterogeneous dose distributions were not prescribed but were accepted as a result of mandatory OAR constraints. Henke et al. used a similar strategy in 5 fractions [25], [26]. They report a mean 70.7% PTV coverage by the 95% isodose line (47.5 Gy) for non-liver abdominal cases in initial plans and suggest a potential improvement in a less hypofractionated regimen [26]. Note also that PTV coverage by the base dose was higher in all dose escalated plans compared to the corresponding base dose plans (both in 5 and 15 fractions). A higher maximum dose constraint (130% of the escalated dose as compared to 130% of the base dose) results in a more heterogeneous dose within the PTV and allows improved coverage by the base dose.

It should be noted that the base dose of BED_10_ = 54 Gy was chosen from previous multi-institutional SBRT series [8]. Whilst being slightly lower than conventional fractionation with 54 Gy in 30 fractions (BED_10_ = 64 Gy), SBRT has been linked to better outcomes than conventional treatments [11]. Possible explanations for this include the radiobiological advantage associated with reduced overall treatment time, as well as the alternative mechanisms of cell kill associated with SBRT such as vascular and immune mediated effects [27]. Shortening the treatment period to 1–3 weeks offers a more convenient schedule to patients, which is particularly relevant given the generally poor prognosis of this population.

A limitation of this study is the equivalence of OAR dose constraints in the two fractionation regimens. The constraints are not BED equivalent (α/β = 3 for GI OAR) but were based on currently recruiting trial protocols [20], [21]. It is acknowledged that there is some uncertainty over the true tolerances of GI OAR and the validity of the linear-quadratic model with high dose per fraction treatments. GI OAR tolerances may be further refined in the future, with the aid of prospectively collected toxicity data from current clinical trials [23], [28]. The 15 fraction constraints were taken from a currently recruiting study evaluating dose escalation in locally advanced biliary tract cancers [21]. Whilst not a pancreas specific protocol, these were pragmatically chosen to reflect the most up-do-date 15 fraction constraints for abdominal hypofractionation. They are in-line with published retrospective series describing clinical outcomes using 15 fraction dose escalated series with acceptable toxicity [12], [29].

Other limitations of this study include the choice of 5 mm isotropic PTV margin. This was deemed sufficient in previous studies of residual liver motion under ABC [18], [19] or pancreatic motion during gating [17], [30]. Using online MR-guidance, gated pancreatic RT with PTV margins of 3 mm has been demonstrated previously [25] and also offers the potential for daily recontouring and replanning in an integrated workflow. A 3 mm margin would allow improved target coverage in general but requires strict motion management under image guidance.

In addition to target motion, OAR motion is an important consideration. Planning at Risk volumes (PRV) are sometimes used to ensure OAR protection [7] but were not used in this study. It is acknowledged that a PRV approach may be preferred when dose escalating to OAR tolerance unless daily adaption is available. This would most likely reduce the achievable coverage, especially by the escalated dose.

Finally, the achievable target coverage by the base dose in 5 fractions and the coverage by the escalated dose in 5 and 15 fractions were all significantly correlated with the percentage of PTV volume overlapping with OAR and the proximity of OAR to the PTV. Interestingly, PTV volume did not show a correlation with target coverage in either fractionation schedule. These correlations are important as overlap and proximity may alter during treatment due to interfraction OAR motion. These changes can be capitalised upon using daily adaptive replanning to increase coverage by the prescription dose on days with favourable anatomy [25]. Using a base dose plan established prior to treatment as a starting point, dose escalation can be optimized online based on the anatomy of the day.

## 5 Conclusions

This study showed that dose escalation to sub-volumes of the PTV is dosimetrically feasible in 5 or 15 fractions for an unselected LAPC population. The proposed strategy to cover the PTV by a base dose with BED_10_ = 54 Gy and maximize coverage by a BED_10_≅100 Gy would be particularly well suited for daily adaptive workflow where dose escalation can be optimized depending on the anatomy of the day.
